# LncRNA TRERNA1 facilitates hepatocellular carcinoma metastasis by dimethylating H3K9 in the CDH1 promoter region via the recruitment of the EHMT2/SNAI1 complex

**DOI:** 10.1111/cpr.12621

**Published:** 2019-04-22

**Authors:** Wei Song, Yuejun Gu, Sen Lu, Huazhang Wu, Zhenxing Cheng, Jiaojiao Hu, Yanyan Qian, Ying Zheng, Hong Fan

**Affiliations:** ^1^ Department of Medical Genetics and Developmental Biology, Medical School of Southeast University, The Key Laboratory of Developmental Genes and Human Diseases, Ministry of Education Southeast University Nanjing China; ^2^ School of Life Science Southeast University Nanjing China; ^3^ The First Affiliated Hospital of Nanjing Medical University Nanjing China; ^4^ Department of Pathophysiology Medical School of Southeast University Nanjing China

**Keywords:** EHMT2, hepatocellular carcinoma, metastasis, TRERNA1

## Abstract

**Objectives:**

Long non‐coding RNAs (LncRNAs) play an important role in hepatocellular carcinoma development, however, as a crucial driver of hepatocellular carcinoma (HCC) metastasis, their functions in tumour metastasis remain largely unknown.

**Materials and methods:**

The lncRNA TRERNA1 expression levels were detected in HCC by quantitative real‐time PCR (qPCR). The function of TRERNA1 was examined by wound‐healing assays, transwell assays and tail vein injection experiments. The potential regulatory mechanisms of TRERNA1 on its target genes were explored by ChIP, RIP, IP and WB assays.

**Results:**

Elevated TRERNA1 levels promoted HCC cell migration and invasion in vitro and in vivo. TRERNA1 recruited EHMT2 to dimethylate H3K9 in the CDH1 promoter region. Furthermore, EHMT2 bound to SNAI1 to suppress CDH1 expression in HCC cells. After inhibiting TRERNA1, the expression level of CDH1 was restored and was involved in the regulation of the EHMT2/SNAI1 complex. The level of TRERNA1 was positively correlated with tumour metastasis and was negatively correlated with the expression of CDH1 in HCC tissues.

**Conclusions:**

For the first time, the current study reveals that TRERNA1 promotes cell metastasis and the invasion of HCC via the recruitment of EHMT2 and/or the EHMT2/SNAI1 complex to suppress CDH1. These data identify a novel mechanism that regulates TRERNA1 in metastatic HCC and provides a potential targeted therapy for HCC patients.

## 1 INTRODUCTION

Hepatocellular carcinoma (HCC) is one of the most common malignancies and is the second leading cause of cancer‐related death.^1^, ^2^, ^3^ Metastatic tumours, rather than primary tumours, are the major cause of death in patients with HCC.^4^ Due to post‐surgical recurrence and metastatic potential, HCC becomes an aggressive malignancy. HCC tumour metastasis is a complex process involving alterations in invasion, dissemination, cancer‐associated vasculature and survival.^5^ A high rate of metastases resulted in a poor prognosis and a high recurrence rate of HCC.^6^ Determining the underlying molecular mechanisms of the metastatic cascade will improve the treatment outcome of metastasis‐targeted therapy.

Long non‐coding RNA (lncRNA), with a minimum length of 200 nucleotides, is an autonomously transcribed RNA that does not encode a protein.^7^ LncRNAs have a unique advantage afforded by their abundance, specificity and spatial conformation complexity for regulating gene expression during the development of various diseases, including tumours.^8^, ^9^, ^10^ Given that lncRNAs can regulate a target gene's transcription by modulating chromatin remodelling, protein scaffolding, promoting RNA decay, ceRNA or enhancer functions, it is likely that they can exhibit different biological functions due to their subcellular localizations.^11^, ^12^ Nuclear‐localized lncRNAs regulate gene expression in *cis* or *trans* by mediating chromosomal conformation,^13^, ^14^ while cytoplasmic lncRNAs are known to modulate the activity or abundance of interacting miRNAs or mRNAs.^15^, ^16^ Several lncRNAs, including ATB, H19, HOTAIR and HULC, are reported to be related to the tumorigenesis and metastasis of HCC.^10^, ^17^, ^18^ LncRNAs may be considered facile regulators for controlling the progression of HCC. Our recent study demonstrated that X protein of Hepatitis B Virus (HBx) elevated the potential oncogenic lncRNA UCA1 to promote HCC by repressing p27Kip1.^19^ However, it is even more difficult to identify a representative lncRNA that plays an important role in the progression of HCC, such as in metastatic HCC.

TRERNA1 was first identified as an enhancer‐like lncRNA.^20^ It was also reported that TRERNA1 stimulated tumour invasion in breast cancer.^21^ Our previous data showed that lncRNAs were correlated with the lymph node metastasis of GC.^22^ Considering that TRERNA1 is involved in the metastasis of multiple tumours, the exploration of the lncRNA TRERNA1 may help to elucidate the mechanism of HCC progression and to evaluate the therapeutic implications for HCC. This will provide new strategies for the diagnosis and treatment of metastatic liver cancer.

## 2 MATERIALS AND METHODS

### 2.1 Cell lines

The immortalized human normal hepatocyte cell line LO2 and the HCC cell lines HepG2, HepG2.215 were purchased from the TCC Cell Bank (Shanghai, China). All cell lines were cultured in RPMI Medium 1640 supplemented with 10% foetal bovine serum, 100 U/mL penicillin and 100 mg/mL streptomycin (Invitrogen, Carlsbad, CA) in 5% CO_2_ at 37°C.

### 2.2 HCC tissue specimens

Hepatocellular carcinoma tissues and paired adjacent non‐tumour tissues were collected from a total of 69 patients who underwent radical resections between 2009 and 2016 at the First Affiliated Hospital with Nanjing Medical University, China. Written‐informed consent was obtained from each patient. Ethical consent was granted from the medical ethics committee of the Medical School of Southeast University.

### 2.3 Plasmid construction and transfection

The sequences of lncRNA TRERNA1 and of the coding gene SNAI1 were cloned into a pcDNA3.1 (+) vector. In addition, vector‐based short hairpin RNAs (shRNAs) against TRERNA1 sequences and scrambled sequences (as control shRNAs) were constructed. These constructs were subsequently transfected into the HepG2 and HepG2.215 cells using Lipofectamine 2000 (Invitrogen, Carlsbad, CA, USA) according to the manufacturer's instructions to generate the stable cell lines HepG2‐pcDNA3.1, HepG2‐TRERNA1, HepG2‐SNAI1, HepG2.215‐ConshRNA, and HepG2.215‐shTRERNA1. The transfected cells were cultured in selection media containing 400 μg/mL G418 (GIBCO, Gaithersburg, MD). Small interfering RNAs (siRNAs) and their respective negative controls were synthesized by GenePharma (Shanghai, China). After transfection with the siRNAs using Lipofectamine 2000 for 48 hours, the RNA or protein of cells was collected for analysis. The sequences of the primers used for plasmid construction and the siRNAs against specific targets in this study are listed in Table S1.

### 2.4 RNA isolation and qRT‐PCR analysis

Total RNA was isolated from frozen tissues or cultured cells using TRIzol Reagent (Invitrogen, Carlsbad, California, USA), and 1 μg of total RNA was reverse‐transcribed using the Reverse Transcription Kit (Takara, Dalian, China). Quantitative real‐time PCR (qRT‐PCR) was performed on samples in triplicate using SYBR Green (Takara, China) with a StepOne Plus system (Applied Biosystems, Foster City, CA) according to standard quantitative PCR protocol. The expression levels of the tested genes were normalized against the β‐actin expression levels as a control by using the 2^‐ΔΔCt^ method. The primer sequences used for qRT‐PCR in this study are listed in Table S2.

### 2.5 Wound‐healing, cell migration and invasion assays

For the wound‐healing assays, cell monolayers were scratched with a clean pipette tip, and cell migration was observed by taking photos under a microscope at 0, 24 and 48 hours after scratching. The cell migration assays were performed using 8.0 mm pore size Transwell inserts (Millipore). The cells (1 × 10^5 ^cells in 200 μL of serum‐free media) were seeded onto the upper surface of the Transwell inserts. A total of 700 μL of complete medium with serum were added to the bottom wells of the chambers. Invasion was performed with Matrigel (BD Biosciences) following the manufacturer's protocol. Matrigel was polymerized in transwell inserts for 30 minutes at 37°C. For both transwell assays, the cells were incubated for 24 hours (migration assay) or for 48 hours (invasion assay) in 5% CO_2_ at 37°C. The cells at the top of the Transwell were removed using a cotton swab, and the cells that had migrated to the bottom of the wells were stained with a crystal violet/methanol solution. Photographs of three randomly selected fields were taken, and the average number of cells was determined. Both types of assays were repeated in triplicate experiments independently.

### 2.6 In vivo xenograft mouse model

Four‐week‐old male athymic BALB/c nude mice (Yangzhou University Medical Center, China) were housed and fed in standard, pathogen‐free conditions. Nude mice were injected with 2 × 10^6^ cells in 0.2 mL phosphate buffer solution (PBS) via lateral tail vein injections (n = 6 per group). The average weight of the mice was measured weekly after the injections. After 8 weeks, the mice were euthanized and the livers were removed immediately. Photographs were taken to count the metastatic nodules. The metastatic tissues were confirmed by histopathological H&E staining analysis. This animal study was approved by the Use Committee for Animal Care of Jiangsu Province. All procedures followed the institutional standard guidelines of the Medical School of Southeast University.

### 2.7 Western blot analysis

Cells were lysed with RIPA lysis buffer and were centrifuged at 18 407 ***g*** at 4°C. The protein concentration was quantified using a bicinchoninic acid (BCA) protein assay kit (Beyotime). Identical quantities of protein were separated by 8 ~ 12% sodium dodecyl sulphate‐polyacrylamide gel electrophoresis and were transferred onto 0.22 μm PVDF membranes (Sigma). After blocking with 5% non‐fat dry milk for 1 hour at room temperature, the membranes were incubated with anti‐E‐cadherin (Abcam), anti‐SNAI1 (R&D) and anti‐β‐actin (Sigma‐Aldrich) as an internal reference. After incubation with the HRP‐conjugated secondary antibodies (Sigma), the proteins were detected using a chemiluminescence scanner (Tanon).

### 2.8 RNA immunoprecipitation (RIP)

RIP assays were conducted using the Magna RIP™ RNA‐Binding Protein Immunoprecipitation Kit (Millipore). The cells were prepared in RIP lysis buffer, magnetic beads were prepared for immunoprecipitation, and the protein‐RNA complexes were immunoprecipitated using the anti‐EHMT2 (ab40542), anti‐SNRNP70 (positive control) or normal rabbit IgG (negative control) antibodies. The coprecipitated RNA was purified and analysed by reverse transcription‐PCR (RT‐PCR) or by quantitative real‐time PCR (qRT‐PCR) analyses. The primers used are listed in Table S2.

### 2.9 Chromatin immunoprecipitation (ChIP)

The cells were prepared using the EZ‐Magna ChIP™ G Immunoprecipitation Kits (Millipore) according to the manufacturer's instructions. Briefly, the samples were cross‐linked with 1% formaldehyde, followed by sonication to make soluble chromatin with DNA fragments between 200 and 700 bp. Immunoprecipitation was conducted with the following ChIP‐grade antibodies: anti‐EHMT2 (ab40542), anti‐H3K9 me2 (#4658, CST), anti‐Pol II (positive control) and normal mouse IgG (negative control). DNA was extracted and was used for RT‐PCR or qRT‐PCR analyses. The specific primers for CDH1 used are listed in Table S2.

### 2.10 Immunoprecipitation assay (IP)

The cell lysates were extracted from cultured HepG2.215 cells at 48 hours. The lysates were separated with centrifugation at 4°C for 15 minutes at 12 000 *g*. The immunoprecipitation assay was performed according to the standard protocol of the Pierce Classic IP Kit (Thermo). Anti‐EHMT2 (Abcam, ab40542) and anti‐SNAI1 (R&D) were used to pull down EHMT2 and SNAI1, respectively. IgG was used as a negative control.

### 2.11 Statistical analysis

Pearson's chi‐square (χ^2^) test was used to analyse the correlation between the TRERNA1 expression and the clinicopathological characteristics. Pearson's correlation coefficient was calculated to determine the correlation between two variables using Origin 8.0 software. An independent Student's *t* test (two‐tailed) was performed by comparing the results between the two groups; the data are presented as the mean ± SD. A *P*‐value < 0.05 was considered statistically significant (*P < 0.05, **P < 0.01). Error bars represent the mean ± SD, and ns means not significant.

## 3 RESULTS

### 3.1 TRERNA1 promotes cell migration and invasion of HCC in vitro

**Figure 1:**
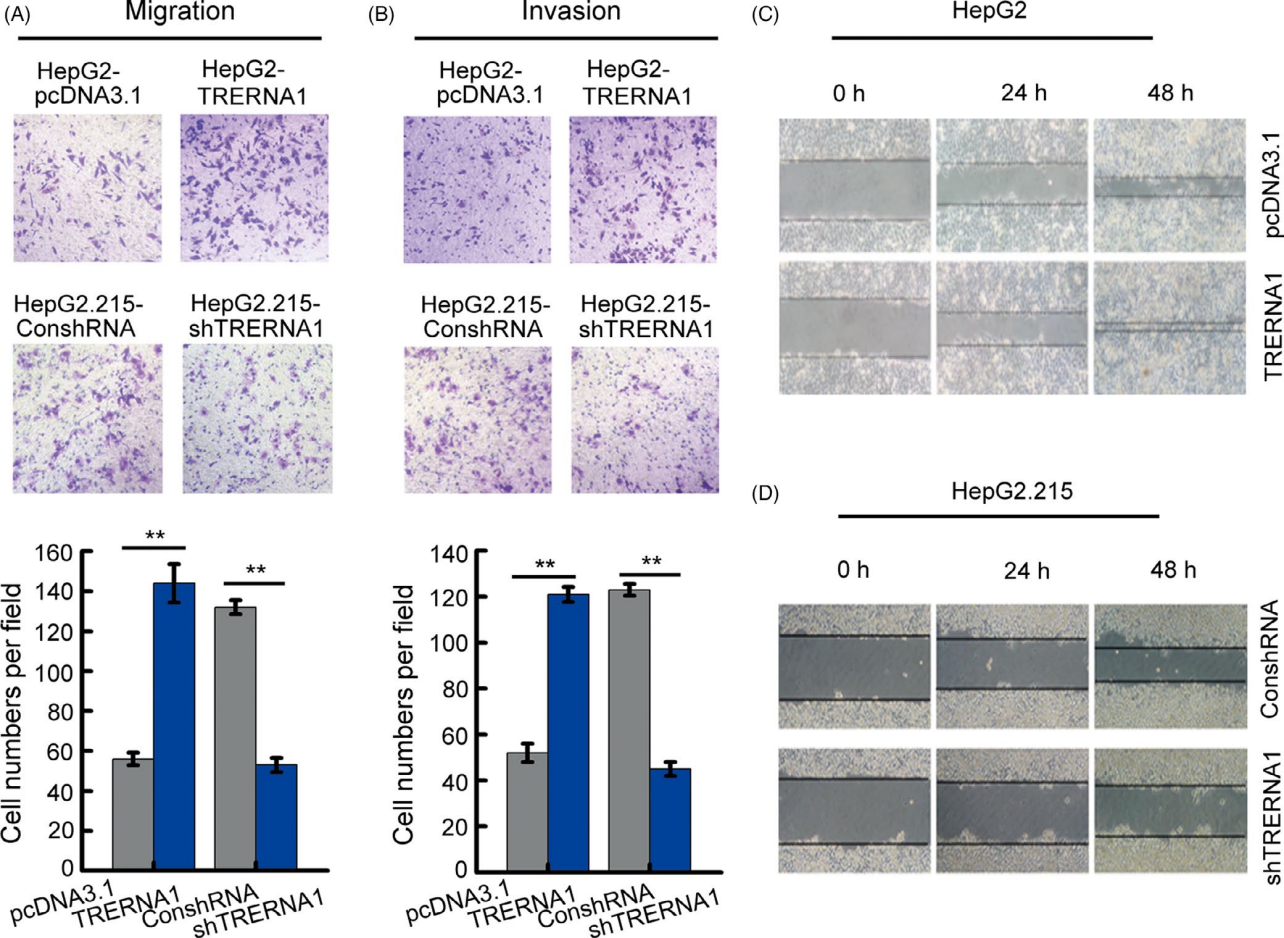
TRERNA1 promotes the cell migration and invasion of HCC in vitro. A, Cell migration assays were performed using transwell assays in HepG2 and HepG2.215 cells. The average number of cells exhibiting migration from three random microscopic fields is presented in the histogram. B, Cell invasion assays were performed using Matrigel‐coated transwell membranes. The average number of cells exhibiting invasion from three random microscopic fields is presented in the histogram. C and D, Wound‐healing assays in TRERNA1‐overexpressing HepG2 cells and in TRERNA1‐depleted HepG2.215 cells. The scratch was measured at 0, 24 and 48 h. Data are presented as the mean ± SD; n = 3. *P < 0.05, **P < 0.01

To further understand the association between TRERNA1 and metastatic HCC, we first examined the function of TRERNA1 on cell migration and invasion. After constructing the HepG2‐TRERNA1 cell line, the HepG2.215‐shTRERNA1 cell line and the corresponding control, the expression levels of TRERNA1 were measured (Figure S1A,B). The exogenous expression of TRERNA1 prompted cell migration and invasion compared with the control cells using both the transwell migration and Matrigel invasion assays. Conversely, the knockdown of TRERNA1 in HepG2.215 cells suppressed cell migration and invasion compared with the control (Figure 1A,B). Similarly, overexpressed TRERNA1 remarkably increased cell mobility in a wound‐healing assay (Figure 1C). In contrast, knockdown of TRERNA1 significantly reduced the wound healing ability of HCC cells (Figure 1D). In other HCC cell lines Huh7 and Hep3B, the ability of cell migration and invasion were also promoted by elevated TRERNA1 level after transiently transfected TRERNA1 or siTRERNA1 in cells, respectively (Figure S2). Taken together, these results demonstrated that TRERNA1 increased the migration and invasion abilities of HCC cells.

### 3.2 TRERNA1 promotes metastasis of HCC cells in vivo

**Figure 2:**
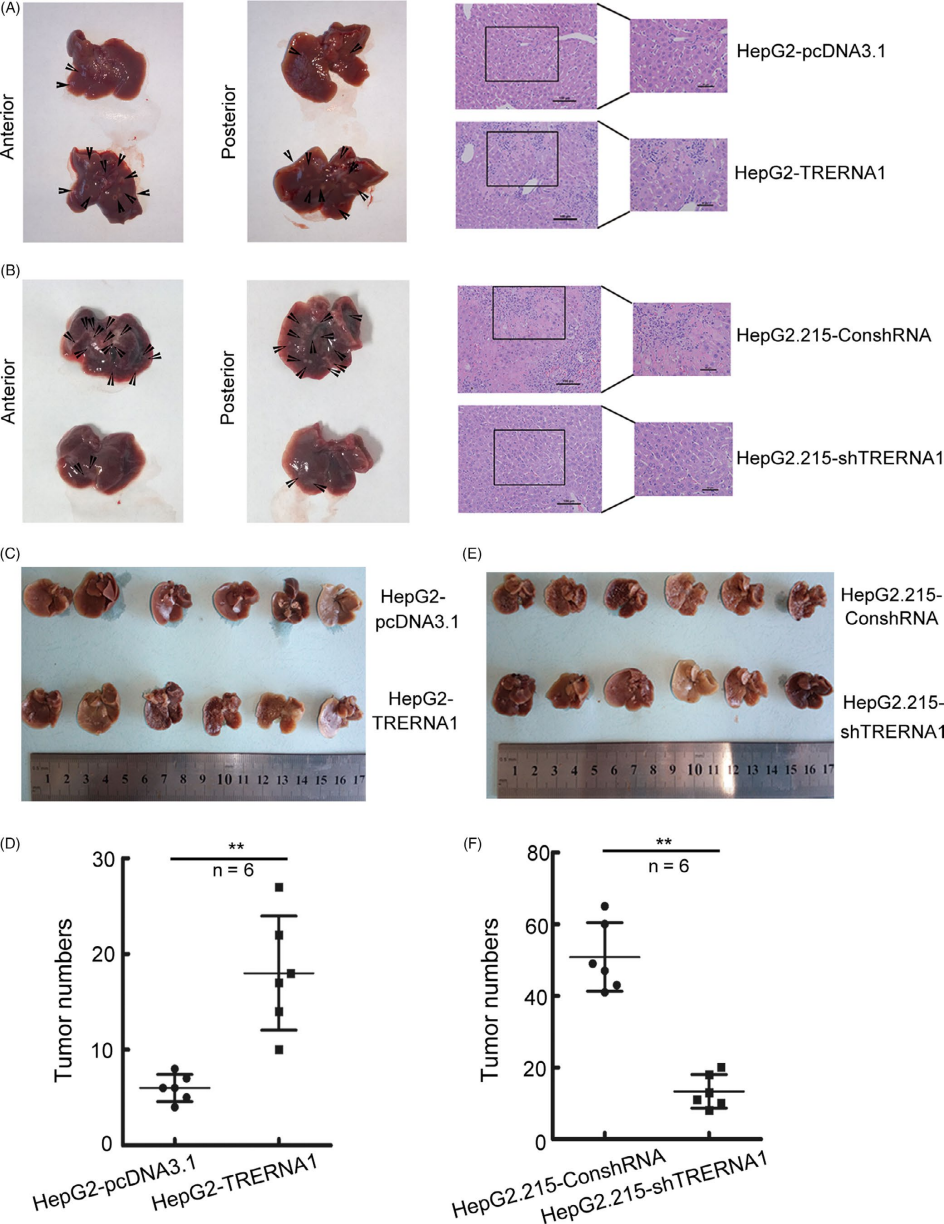
TRERNA1 promotes the metastasis of HCC cells in vivo. A and B, Representative livers derived from severe combined immunodeficient (SCID) mice after tail vein injection with HepG2‐TRERNA1 and HepG2.215‐shTRERNA1 were shown. Haematoxylin and eosin‐stained (H&E) images of liver tissues isolated from the mice were shown. Scale bars represent 500 µm (left) and 100 µm (right). Arrows indicate metastasis nodules. C and D, Ectopic TRERNA1 expression promoted the metastasis of HepG2 cells in vivo, and the number of liver metastasis nodules in the mice was determined after 8 weeks (n = 6 per group). E and F, The knockdown of endogenous TRERNA1 by shRNA inhibited the liver metastasis nodules of HepG2.215 cells in nude mice (n = 6 per group). Data are presented as the mean ± SD; **P < 0.01 (Student's *t* test)

Subsequently, we evaluated the function of TRERNA1 in promoting HCC metastatic capacity in vivo*.* We established a liver metastatic model in nude mice by tail vein injections. After injection with HepG2‐TRERNA1 or HepG2.215‐shTRERNA1 in liver metastatic model mice or control cells for 8 weeks, the mice were euthanized, and liver metastatic nodules were counted. More micrometastases were observed in the TRERNA1‐overexpression group than those in the control group (Figure 2A). More intrahepatic tumour metastases were observed in the HepG2‐TRERNA1 group (Figure 2C,D). In contrast, few metastatic nodules were observed in the livers of the HepG2.215‐shTRERNA1 group (Figure 2B, E and F). Overall, our data supported that TRERNA1 played an important role in the promotion of HCC metastasis in vivo.

### 3.3 TRERNA1 represses CDH1 expression by recruiting EHMT2 to dimethylate H3K9 in the CDH1 promoter region

**Figure 3:**
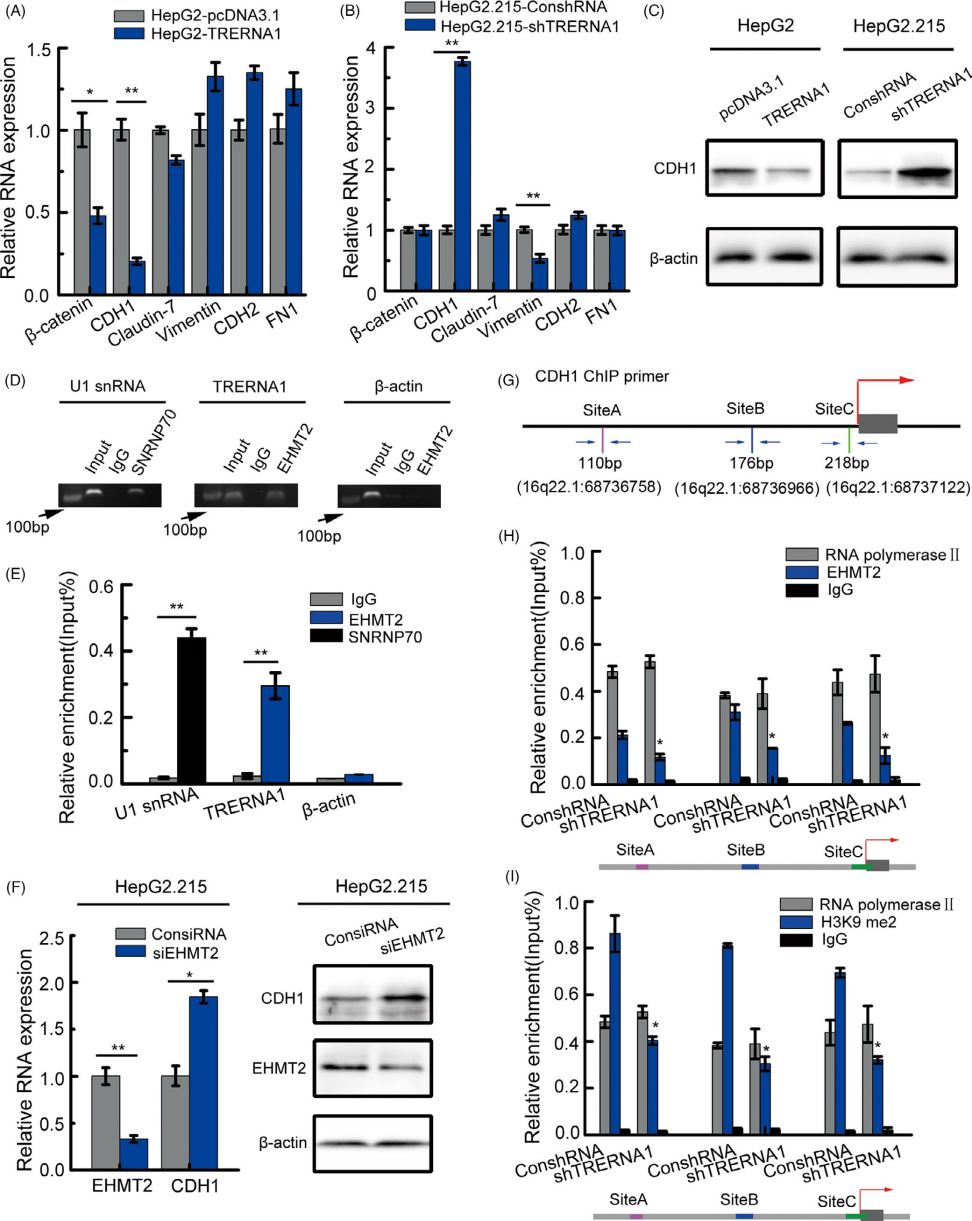
TRERNA1 functions as a scaffold to suppress CDH1 expression by recruiting EHMT2. A, The relative mRNA expression levels of epithelial and mesenchymal markers were measured in TRERNA1‐overexpressing HepG2 cells. B, The relative mRNA expression levels of epithelial and mesenchymal markers were measured in TRERNA1‐depleted HepG2.215 cells. C, Western blot analysis of CDH1 expression in HepG2 cells transfected with TRERNA1 or pcDNA3.1. Western blot analysis of CDH1 expression in HepG2.215 cells transfected with shTRERNA1 or the control. D, The gel electrophoresis results of the PCR products from the RIP assay of the enrichment of EHMT2 on TRERNA1 in HepG2.215 cells was shown. SNRNP70 and IgG were used as positive and negative controls, respectively. E, TRERNA1, U1 snRNA and β‐actin from the RIP assay were also analysed by qRT‐PCR. C, The relative mRNA level of CDH1 was detected by qRT‐PCR after knocking down EHMT2 in HepG2.215 cells. F, The relative protein level of CDH1 was detected by a Western blot after knocking down EHMT2 in HepG2.215 cells. G, Schematic diagram showed the primer position of 3 detection sites (A, B and C) on the CDH1 promoter by ChIP. H, Anti‐EHMT2 ChIP assay and qRT‐PCR were employed to detect the binding of EHMT2 on the CDH1 promoter region after the inhibition of TRERNA1 in HepG2.215 cells. I, Anti‐H3K9me2 ChIP assay and qRT‐PCR were performed to detect the enrichment of H3K9me2 on the CDH1 promoter region after the knockdown of TRERNA1 in HepG2.215 cells. RNA polymerase II and IgG were used as positive and negative controls in (H, I), respectively. Data are shown as the mean ± SD; n = 3. *P < 0.05, **P < 0.01 (Student's *t* test)

To explore the mechanism of the TRERNA1 stimulation of HCC metastasis, we evaluated the impact of TRERNA1 on metastasis‐related genes, especially the epithelial‐mesenchymal transition (EMT)‐related genes at the transcript level (Figure 3A,B). The predominately altered gene in HepG2‐TRERNA1 and/or HepG2.215‐shTRERNA1 is CDH1, which encodes E‐cadherin to maintain the morphology of epithelial cells. When transiently transfected TRERNA1 in Huh7 cells, we observed the reduction in CDH1 level. Otherwise, knocked‐down TRERNA1 increased the expression level of CDH1 in Hep3B cells (Figure S3). We also noted that the loss of function of E‐cadherin often occurs in migratory cells and is related to tumour metastasis.^23^ In the present study, we found that elevated TRERNA1 decreased the expression level of CDH1 by Western blotting; conversely, the CDH1 expression level was restored after the knockdown of TRERNA1 (Figure 3C; Figure S4A,B). The mechanism by which TRERNA1 induces CDH1 in these cells was further investigated.

Emerging studies have revealed that lncRNAs play divergent roles in epigenetic regulatory networks.^13^, ^24^ Some lncRNAs regulate gene expression by histone methylation via recruiting chromatin regulatory complexes.^25^, ^26^, ^27^ EHMT2, a major euchromatin methyltransferase responsible for H3K9me2 methylation, can recruit transcription factors during cell biological processes.^28^, ^29^ In an RNA‐binding protein immunoprecipitation (RIP) assay, we found a potential physical interaction between TRERNA1 and EHMT2 (Figure 3D). Subsequently, RIP‐qRT‐PCR was employed to confirm the binding between TRERNA1 and EHMT2 compared with the binding in IgG controls (Figure 3E). After knocking down EHMT2, the expression level of CDH1 was increased (Figure 3F; Figure S4C). In addition, the reduced enrichment of EHMT2 in the CDH1 promoter region was also observed when TRERNA1 expression was decreased (Figure 3G,H). To investigate the potential mechanism by which TRERNA1 inhibits CDH1, an antibody against H3K9me2 was used for ChIP assays. We observed that the inhibition of TRERNA1 reduced the H3K9 dimethylation levels in the CDH1 promoter region (Figure 3I). These results suggested that TRERNA1 epigenetically silenced CDH1 by altering its H3K9me2 levels *in trans*, which depended on EHMT2.

### 3.4 TRERNA1 recruits EHMT2 for binding with SNAI1 to repress CDH1 expression

**Figure 4:**
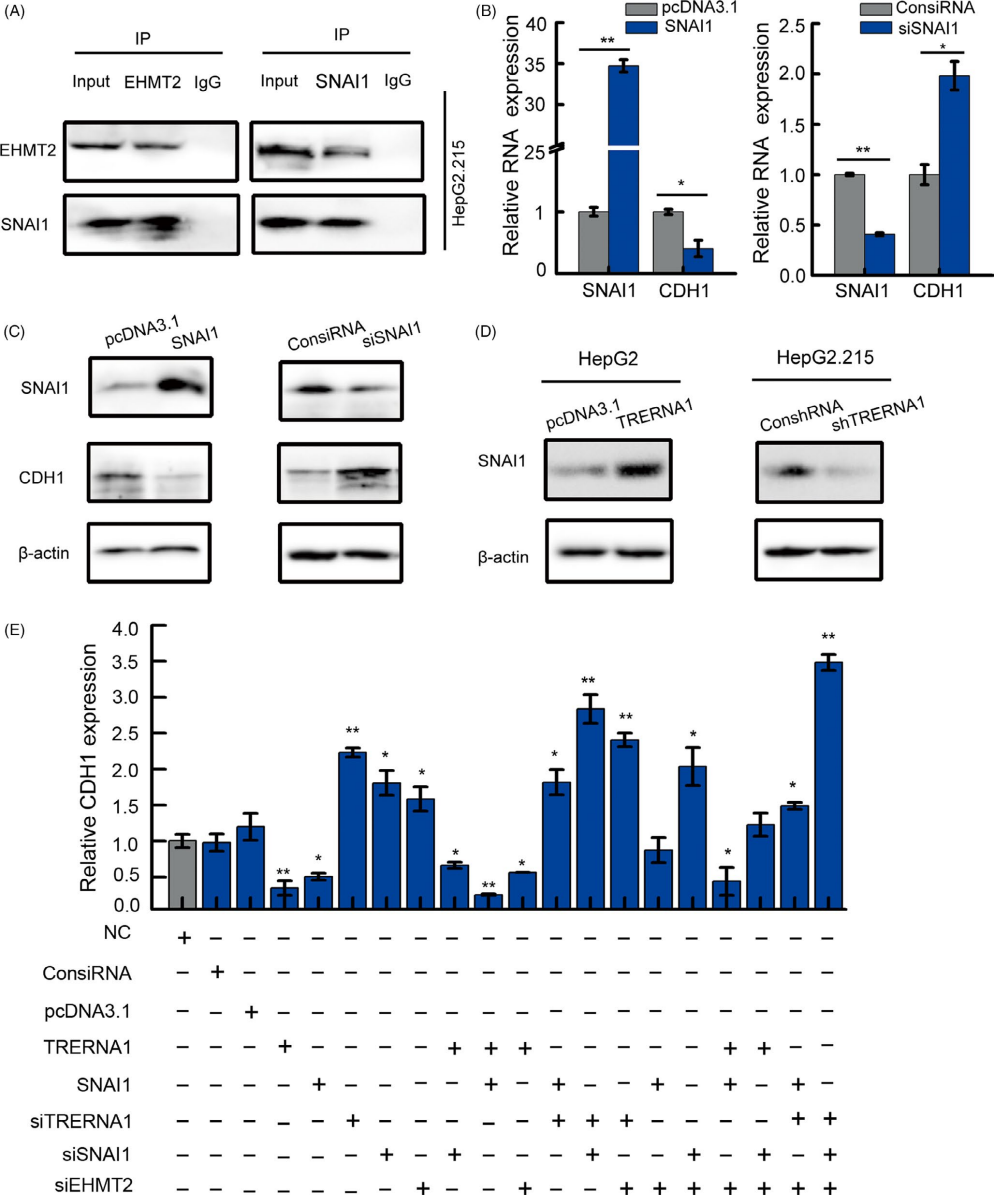
TRERNA1 recruits EHMT2 binding with SNAI1 to repress CDH1 expression. A, Immunoprecipitation of endogenous EHMT2 and its associated endogenous SNAI1 was analysed by Western blot. B, The CDH1 expression level was analysed by qRT‐PCR in HepG2/HepG2.215 cells after transfection with SNAI1 or siSNAI1. C, After the overexpression or knockdown of SNAI1, the CDH1 expression was detected by a Western blot in HepG2/HepG2.215 cells. D, After the overexpression or knockdown of TRERNA1, the SNAI1 expression levels were analysed by a Western blot analysis. E, The mRNA levels of CDH1 were analysed by qRT‐PCR in HepG2 cells transfected with ConsiRNA, pcDNA3.1, TRERNA1, siTRERNA1, SNAI1, siSNAI1 and siEHMT2. Data are shown as the mean ± SD; n = 3. *P < 0.05, **P < 0.01 (Student's *t* test)

In the following immunoprecipitation assays, we found that EHMT2 coprecipitated with SNAI1, a cell migration‐related transcriptional factor (Figure 4A). SNAI1 is a vital regulator of cell adhesion, migration, invasion and EMT.^30^ In our study, the coprecipitation of SNAI1 and EHMT2 was involved in the regulation of TRERNA1 on the expression of E‐cadherin encoded by CDH1. Thus, we first examined the effect of SNAI1 on HCC metastasis/CDH1 in our model. SNAI1 mRNA levels also inhibited CDH1 expression (Figure 4B). We also found that the reduction in SNAI1 increased the expression of CDH1 at the protein level, while the overexpression of SNAI1 resulted in a reciprocal change (Figure 4C; Figure S4D‐E). Surprisingly, TRERNA1 also regulated the protein expression of SNAI1 (Figure 4D; Figure S4F). These data indicate, at least in our model, that TRERNA1 also plays an important role in the regulation of SNAI1, which suggests that the presence of TRERNA1 has an epigenetic role in the regulation of SNAI on CDH1.

To investigate the underlying mechanism whereby TRERNA1 silences CDH1 by binding to EHMT2/SNAI1, the CDH1 expression levels were observed using different treatments with TRERNA1, SNAI1 and EHMT2, taking the EHMT2 coprecipitate with SNAI1 into consideration. As shown in Figure 4E (bar 10), the overexpression of both TRERNA1 and SNAI1 significantly reduced the CDH1 expression in HepG2.215 cells. More interestingly, compared with silenced SNAI1 and EHMT2 only (Figure 4E, bar 16), knocking down TRERNA1 again in this model resulted in a significantly higher CDH1 level (Figure 4E, bar 20). These results demonstrated that TRERNA1 recruited EHMT2 and/or formed EHMT2/SNAI1 complexes that repressed the CDH1 expression in HCC cells.

### 3.5 TRERNA1 promotes the ability of SNAI1 to treat HCC metastasis

**Figure 5:**
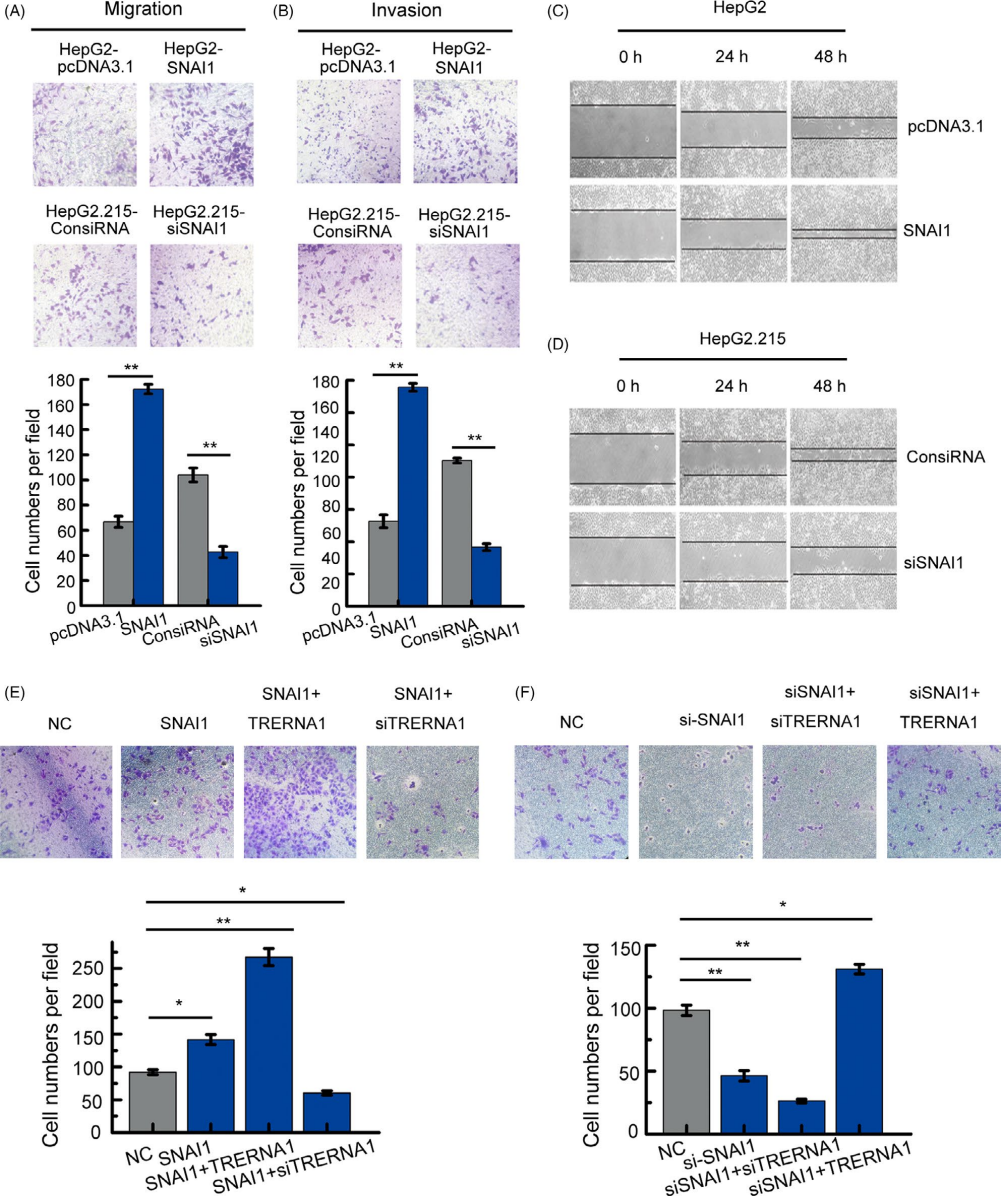
TRERNA1 promotes the ability of SNAI1 to treat HCC metastasis. A, Cell migration assays were performed using transwell membranes. The average number of cells exhibiting migration from three random microscopic fields is presented in the histogram. B, Cell invasion assays were performed using Matrigel‐coated transwell membranes. The average number of cells exhibiting invasion from three random microscopic fields is presented in the histogram. C and D, A wound‐healing assay in SNAI1‐overexpressing HepG2 cells and in SNAI1‐depleted HepG2.215 cells. The scratch was measured 0, 24 and 48 h later. E, Cell migration assays using transwells in HepG2 cells treated with the ctrl, SNAI, SNAI + TRERNA1, or SNAI + siTRERNA1. The average number of cells exhibiting migration from three random microscopic fields was presented in the histogram. F, Cell migration assays using transwells in HepG2.215 cells treated with the ctrl, siSNAI1, siSNAI + siTRERNA1, or siSNAI1 + TRERNA1. The average number of cells exhibiting migration from three random microscopic fields is presented in the histogram. The results are presented as the mean ± SD; n = 3. *P < 0.05, **P < 0.01 (Student's *t* test)

In our model, SNAI1 is involved in the regulation of CDH1 by TRERNA1; thus, we considered the effect of SNAI1 on the metastatic ability of HCC. Our results indicated that SNAI1 increased the ability of cells to migrate and invade (Figure 5A,B). Similarly, SNAI1 remarkably increased the wound healing ability of HCC cells (Figure 5C,D). To investigate the relationship between TRERNA1 and SNAI1 in the process of HCC migration, HepG2 cells were transfected with SNAI1, SNAI1 + TRERNA1, SNAI1 + siTRERNA1 or control (ctrl), and HepG2.215 cells were treated with siSNAI1, siSNAI1 + siTRERNA1, siSNAI1 + TRERNA1 or control (ctrl). In a transwell assay, we found that overexpressed SNAI1 promoted cell migration; however, knocking down TRERNA1 reduced the migration ability of HepG2‐SNAI1 cells (Figure 5E). Otherwise, the silencing of SNAI1 blocked cell migration in HepG2.215 cells, whereas the induction of TRERNA1 expression, at least partially, rescued cell migration (Figure 5F). Taken together, these results demonstrated that TRERNA1 enhanced the metastatic ability of SNAI1 in HCC cells.

### 3.6 Overexpressed TRERNA1 levels are correlated with metastasis and are negatively correlated with tumour metastasis repressor gene CDH1 expression in HCC patients

**Figure 6:**
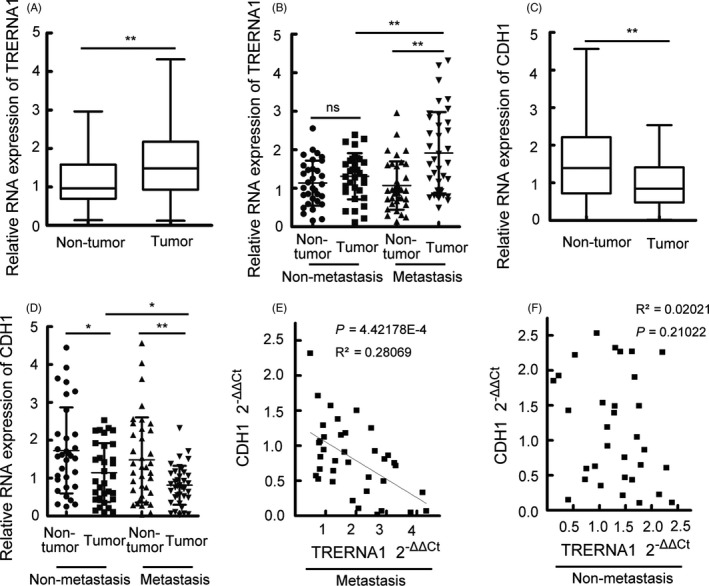
Overexpressed TRERNA1 levels are correlated with metastasis and are negatively correlated with tumour metastasis repressor gene CDH1 expression in HCC patients. A, The expression levels of TRERNA1 in HCC tissues and in paired, adjacent non‐tumour tissues were analysed by qRT‐PCR. The horizontal lines in the box plots represent the medians, the boxes represent the interquartile range, and the whiskers represent the 2.5th and 97.5th percentiles. The significant differences were analysed by the Wilcoxon signed‐rank test. B, The TRERNA1 expression level in HCC metastasis tissues (n = 37) and in non‐metastatic tissues (n = 32). β‐actin was used as an internal control. C, The expression level of CDH1 in HCC tissues and in paired adjacent non‐tumour tissues by qRT‐PCR (n = 69). The horizontal lines in the box plots represent the medians, the boxes represent the interquartile range, and the whiskers represent the 2.5th and 97.5th percentiles. The significant differences were analysed by the Wilcoxon signed‐rank test. D, CDH1 expression levels were examined in HCC metastasis tissues (n = 37) and in non‐metastatic tissues (n = 32). E, The correlation between the CDH1 mRNA level and the TRERNA1 transcript level was measured by qRT‐PCR in metastatic tumour tissues. The 2^‐ΔΔCt^ values (normalized to β‐actin) were subjected to Pearson's correlation analysis (*P* = 4.42178E‐4, *R* ^2 ^= 0.28069). F, The correlation between the CDH1 mRNA level and the TRERNA1 transcript level was measured by qRT‐PCR in non‐metastatic tumour tissues. The 2^‐ΔΔCt^ values (normalized to β‐actin) were subjected to Pearson's correlation analysis (*P* = 0.21022, *R* ^2 ^= 0.02021). Data are presented as the mean ± SD; n = 3. *P < 0.05, **P < 0.01

To investigate the potential role of TRERNA1 in human HCC, we examined the levels of TRERNA1 in 69 HCC tumour tissues and their paired, adjacent non‐tumour tissues. The data showed that TRERNA1 demonstrated higher expression levels in the tumour tissues compared with that in the pair‐matched adjacent non‐tumour tissues (Figure 6A). In clinical HCC tissues, qRT‐PCR analysis of TRERNA1 expression in 69 cases showed that TRERNA1 was overexpressed in 53% of HCC tissues (Figure S5A). Next, we evaluated the correlation between TRERNA1 mRNA expression levels and clinicopathological characteristics in HCC patients (Table 1). Kaplan‐Meier analysis showed that high levels of TRERNA1 were significantly associated with tumour metastasis (*P* = 0.021), although there were no remarkable differences between the higher TRERNA1 level group and the lower one in the patients’ gender, age and HBs antigen (Table 1). Moreover, we observed that high TRERNA1 expression levels were positively correlated with metastatic tumours (Figure 6B). All these data showed that the aberrant upregulation of TRERNA1 was correlated with metastasis in HCC, which indicates that TRERNA1 may play an important role in the progression and prognosis of HCC.

**Table 1:** Clinicopathological correlation of TRERNA1 expression level in HCC cases

Feature	TRERNA1 expression		χ^2^	*P* value
	Low	High		
All cases	35	34		
Gender			0.097	0.756
Male	30	30		
Female	5	4		
Age			1.365	0.243
≥60	7	11		
<60	28	23		
Tumours size			0.414	0.520
>4 cm	20	22		
≤4 cm	15	12		
Edmondson‐Steiner grade			1.740	0.187
Ⅰ/Ⅱ	22	16		
Ⅲ/Ⅳ	13	18		
Metastasis and invasion			5.301	0.021^a^
Absent	21	11		
Present	14	23		
HBs antigen			0.076	0.782
Positive	29	29		
Negative	6	5		

The median expression level was used as the cut‐off.

HCC, hepatocellular carcinoma

a For analysis of correlation between the expression levels of long non‐coding RNA TRERNA1 and clinical features of HCC, Pearson chi‐square tests were used. Results were considered statistically significant at *P* < 0 0.05.

qRT‐PCR was used to investigate the correlation between TRERNA1 and CDH1 expression in HCC clinical cases. Compared with that in the para‐carcinoma tissues, the CDH1 level was decreased in the HCC tumour specimens (Figure 6C). We also observed that the CDH1 expression level was significantly reduced in metastatic tumour tissues compared with that in non‐metastatic tissues (Figure 6D). In addition, Spearman correlation coefficient analysis showed that there was a significant negative correlation between TRERNA1 and CDH1 in metastatic tumour cases (Figure 6E) but not in non‐metastatic cases (Figure 6F). In an in vivo assay, we monitored the Cdh1 expression levels in nude mice after the injection of HepG2‐TRERNA1 cells (Figure S5B). A tail vein injection of HepG2.215‐shTRERNA1 cells resulted in a higher expression of Cdh1 than the negative controls (Figure S5C). We also found increased expression level of Snai1 in injected HepG2‐TRERNA1 cells group and decreased expression level of Snai1 in injected HepG2.215‐shTRERNA1 cells group (Figure S5D‐E). Taken together, the results showed that elevated TRERNA1 negatively regulated CDH1, which promoted HCC progression.

## 4 DISCUSSION

Metastatic liver cancer is often more resistant to post‐surgical therapy than primary cancers. The mortality and recurrence rates of HCC patients are primarily due to the high metastatic potential.^31^, ^32^ It was generally believed that the occurrence of metastasis, a complex process, is ascribed to the regulation of the gene expression of multiple genes.^4^ Therefore, elucidating the intricate molecular regulatory mechanisms implicated in HCC metastasis is critical for controlling prognosis.^33^

Further insight into the aspects of metastasis can be gleaned by considering the roles of coding genes such as E‐cadherin and PTEN.^34^ In addition, several non‐coding molecules, especially lncRNAs, have been examined for their multiple functions during tumorigenesis. In contrast to other molecules, lncRNAs display diverse regulatory effects according to their complex space conformations.^35^, ^36^, ^37^ The formation of secondary and tertiary structures allows lncRNA to interact with proteins as well as DNA or RNA.^37^ Thus, exploring the regulatory mechanisms of lncRNAs will provide new ideas for the diagnosis and treatment of metastatic liver cancer.

A recent study showed that lncRNAs play a vital role in controlling tumour progression. The lncRNAs ATB, HAND2‐AS1 and FTX have been demonstrated to be related to tumour metastasis.^10^, ^17^, ^38^ However, due to the complexity of the structures and functions of non‐coding RNAs, the exploration of lncRNAs in HCC is far from over. In our study, we report for the first time that the overexpression of TRERNA1 was significantly associated with tumour clinicopathological metastasis characteristics in HCC. In in vitro and in vivo assays, we found that TRERNA1 promoted HCC cell metastasis. Overall, these results convincingly indicated that TRERNA1 plays an important oncogenic role in promoting HCC metastasis.

We detected several markers associated with metastasis and determined whether they were affected by TRERNA1. In particular, E‐cadherin, encoded by CDH1, was a candidate because it not only is the most prominent inducer of EMT in HCC^39^ but also is directly correlated with a poor prognosis and short survival rates for HCC patients.^40^, ^41^ Elevated TRERNA1 regulated the tumour metastasis‐related gene CDH1 expression, although the expression level of β‐catenin and vimentin was also changed in enforced or knocked‐down TRERNA1 cells, respectively. However, the expression of vimentin did not have a significantly change when overexpressed TRERNA1 in HepG2 cells, which suggested that TRERNA1 has no a significant broad regulation on vimentin. What's more, at the translation level, we did not detect a significant expression change of β‐catenin and vimentin regulated by TRERNA1. Thus, CDH1 was considered as a potential molecule for studying the mechanism of TRERNA1. Although CDH1 translation was affected by TRERNA1 in breast cancer,^21^ it is still unclear whether metastasis‐related genes including CDH1 are regulated by TRERNA1 at the transcriptional level. Surprisingly, we first observed that the mRNA levels of CDH1 were changed in TRERNA1‐transfected HCC cells. Subsequently, we found that TRERNA1 recruited EHMT2 to dimethylate H3K9 in the CDH1 promoter region. Given that lncRNAs are involved in histone methylation modification, they have become important epigenetic regulatory molecules during the regulation of target genes.^25^ EHMT2, a major histone methyltransferase for H3K9 me2, is a crucial modifying factor that is regulated during gene silencing. These results encourage us to investigate the potential mechanism of TRERNA1‐mediated coding gene silencing. After knocking down TRERNA1, we observed a decrease in the H3K9me2 and EHMT2 levels in the CDH1 promoter. In ChIP and co‐IP assays, we found that SNAI1 acts as a repressor of CDH1 and binds to EHMT2, which indicates that EHMT2 decreases CDH1 expression not only by dimethylation but also by interacting with SNAI1. In a transwell assay, TRERNA1 enhanced the metastatic ability of SNAI1 in HCC cells. These data suggest that TRERNA1 indeed regulates the CDH1 gene at the transcriptional level in HCC cells. TRERNA1 may play a far more crucial and higher priority function than SNAI1 and EHMT2 in regulating CDH1. In clinical samples, further analysis demonstrated that there was a significant negative correlation between TRERNA1 and CDH1 in metastatic tumour cases. A comprehensive understanding of the mechanism by which TRERNA1 regulates the coding genes through multiple regulatory pathways will be helpful for the treatment of metastatic tumours, including HCC.

**Figure 7:**
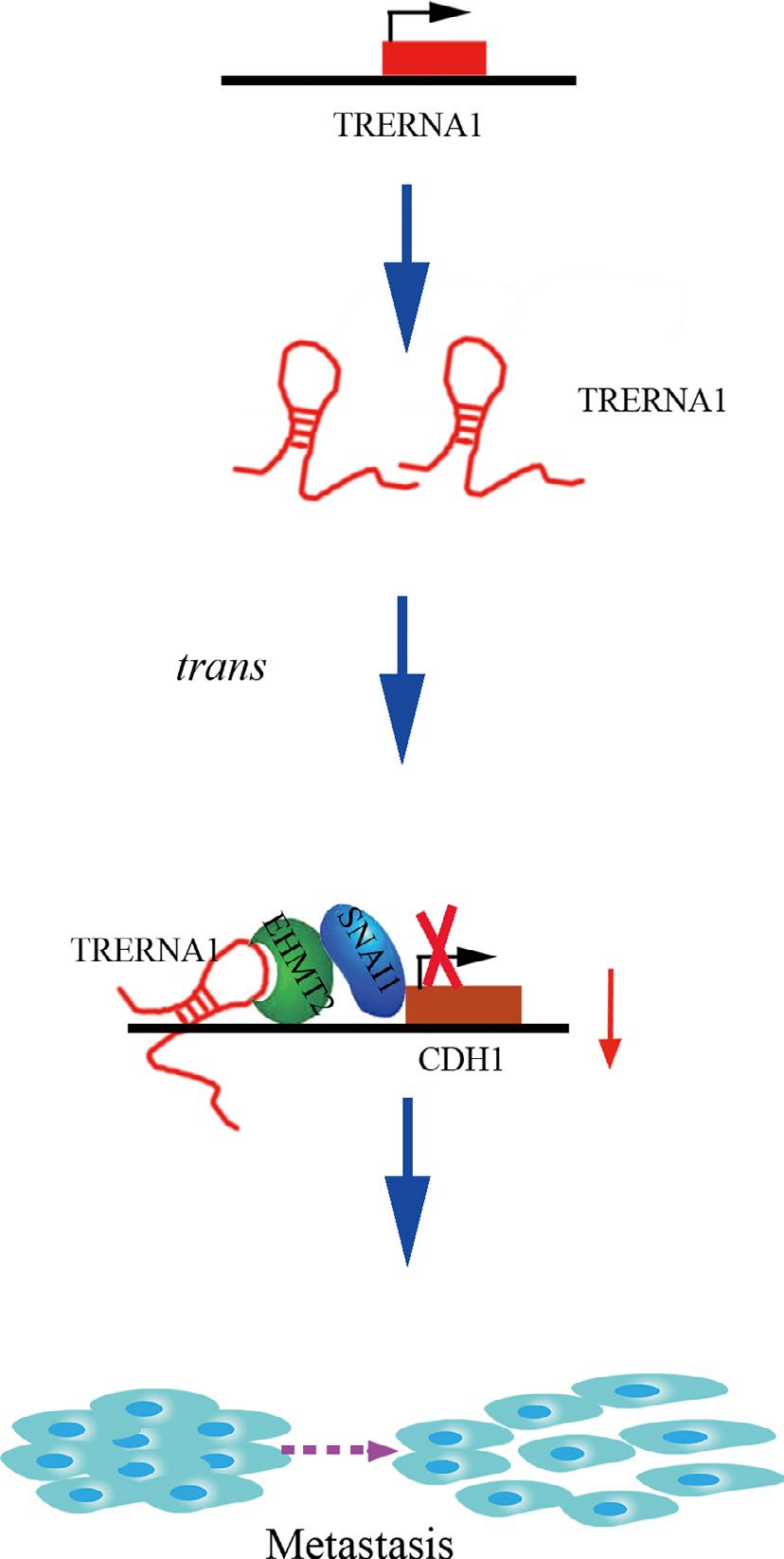
A schematic diagram of the lncRNA TRERNA1 functions in HCC metastasis. LncRNA TRERNA1, which could be upregulated in HCC, recruits EHMT2 as a scaffold and forms an EHMT2/SNAI1 complex to suppress the expression of CDH1 via the dimethylation of H3K9 in promoting HCC metastasis

In summary (Figure 7), we demonstrated that the elevated lncRNA TRERNA1 levels promoted HCC cell metastasis in vitro and in vivo; the aberrant expression of TRERNA1 relates to metastatic HCC and a poor prognosis for patients. TRERNA1 suppresses CDH1 with epigenetic histone modifications via the recruitment of EHMT2 and/or the EHMT2/SNAI1 complex*.* Therefore, targeting the lncRNA TRERNA1 might be a novel therapeutic strategy for metastatic HCC.

## CONFLICT OF INTEREST

The authors declare no competing financial interests.

Consent for publication: Consent to publish has been obtained from all authors.

## AUTHORS’ CONTRIBUTIONS

HF conceived and designed the experiments. WS conducted the experiments and wrote the manuscript. HZW constructed the plasmids used in this study. JJH performed the experiments and analysis of data. SL provided the precious HCC clinical specimens. ZXC performed the animal experiment. YJG and Y.Z collected the clinical sample. YYQ analysed part of data in HCC cell lines. All authors reviewed the manuscript before submission.

## ETHICS APPROVAL AND CONSENT TO PARTICIPATE

The research protocol was reviewed and approved by the Human Research Ethics Committee of Zhongda Hospital, and written‐informed consent was obtained from each patient included in the study.

## DATA AVAILABILITY STATEMENT

All data generated or analysed during this study are included in this published article and its additional files.
