# Supplementary figures and images for: LncRNA TRERNA1 facilitates hepatocellular carcinoma metastasis by dimethylating H3K9 in the CDH1 promoter region via the recruitment of the EHMT2/SNAI1 complex
Source: Cell Prolif. 2019 Apr 22;52(4):e12621. doi: 10.1111/cpr.12621 (PMC6668973; doi:10.1111/cpr.12621)

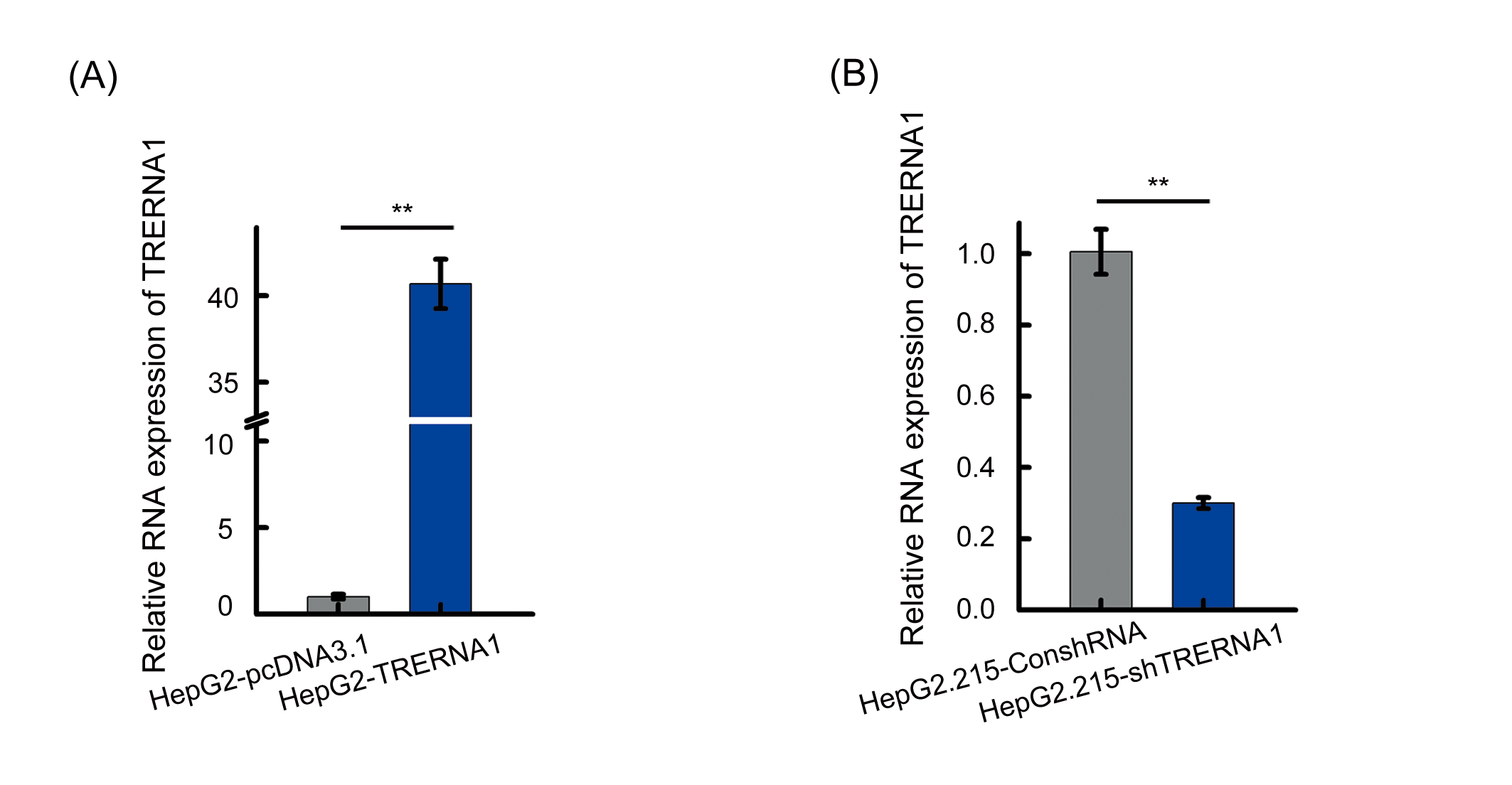

Supplement: Supplementary file 1 [file CPR-52-e12621-s001.tif]

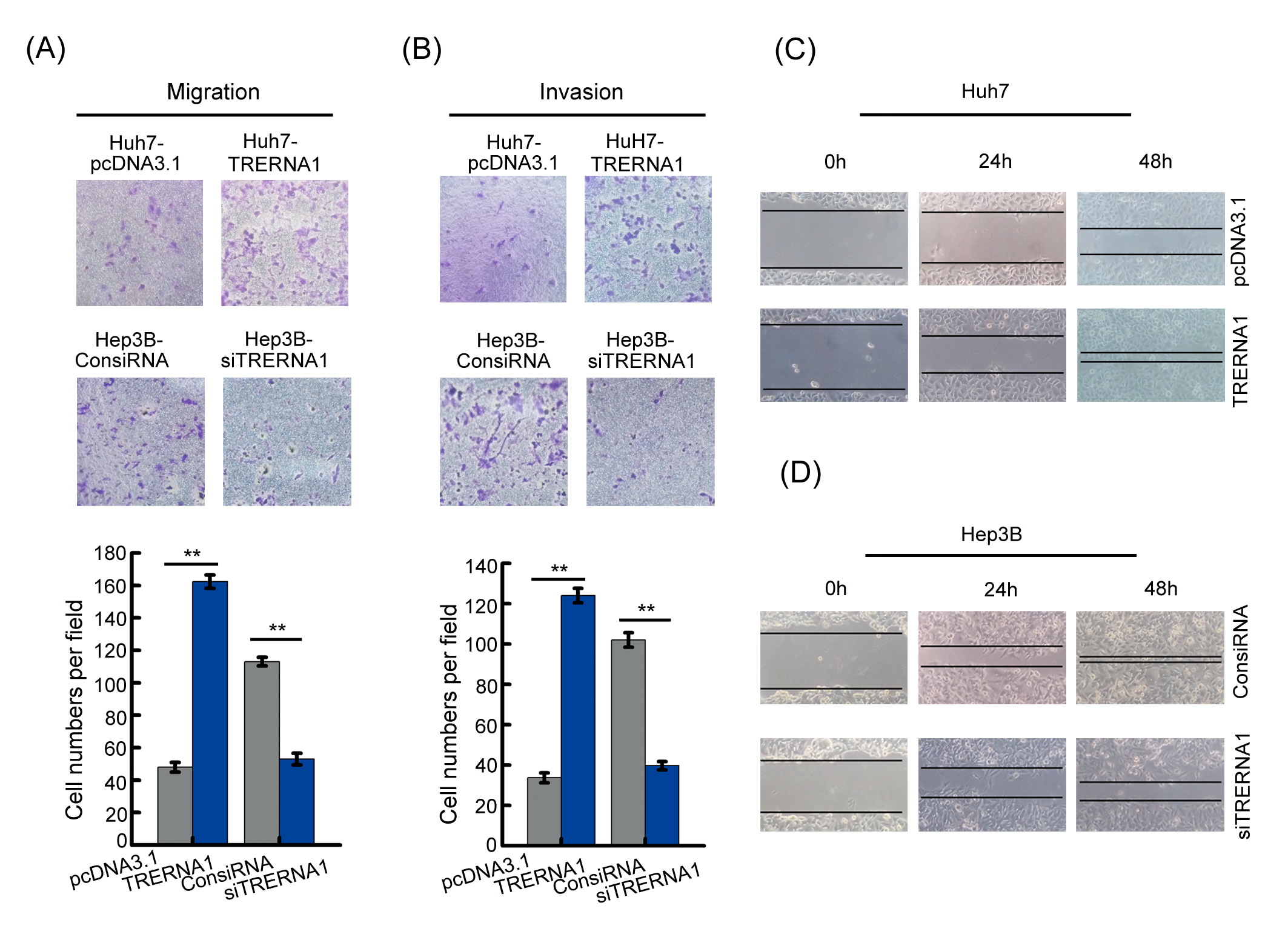

Supplement: Supplementary file 2 [file CPR-52-e12621-s002.tif]

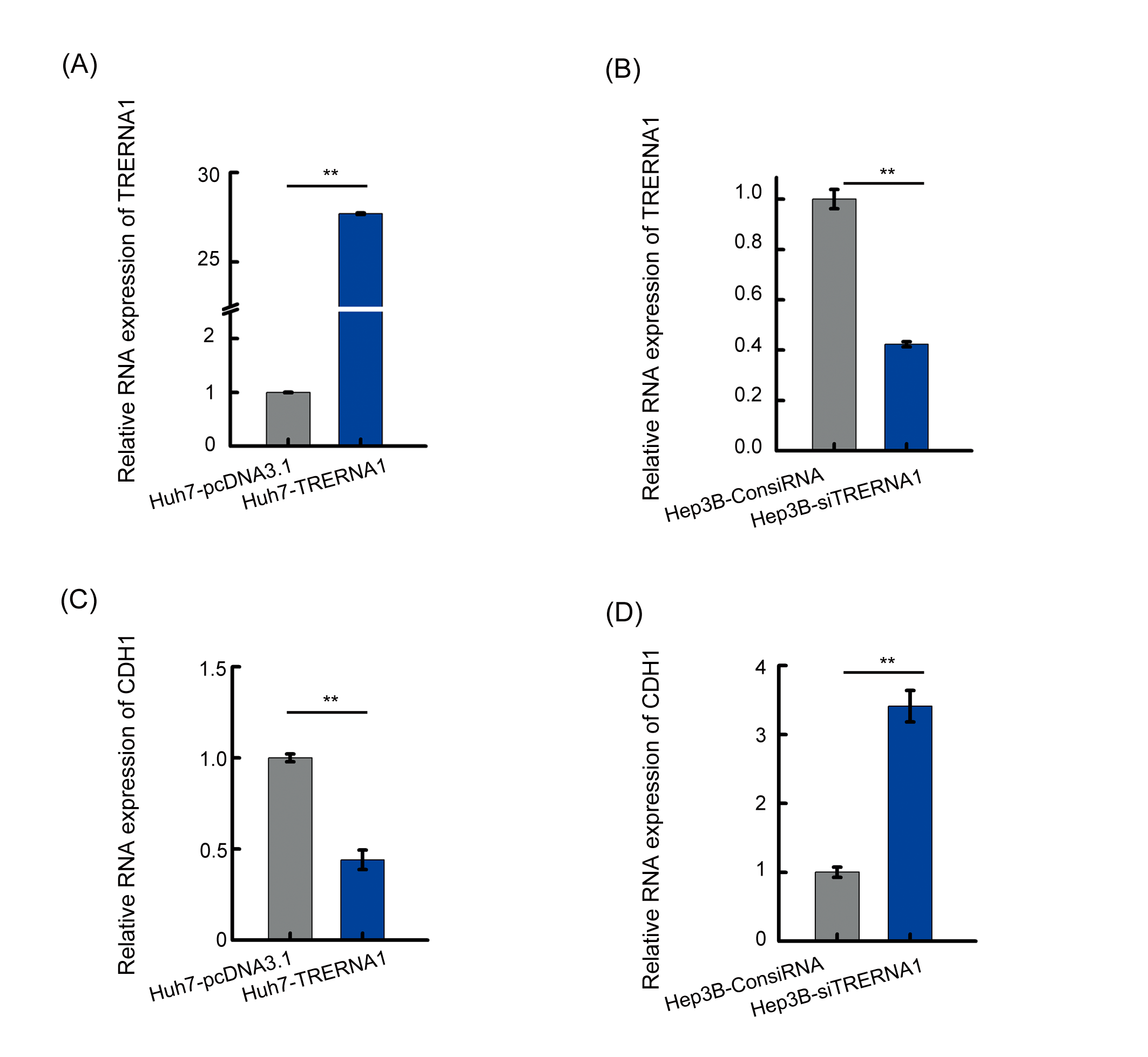

Supplement: Supplementary file 3 [file CPR-52-e12621-s003.tif]

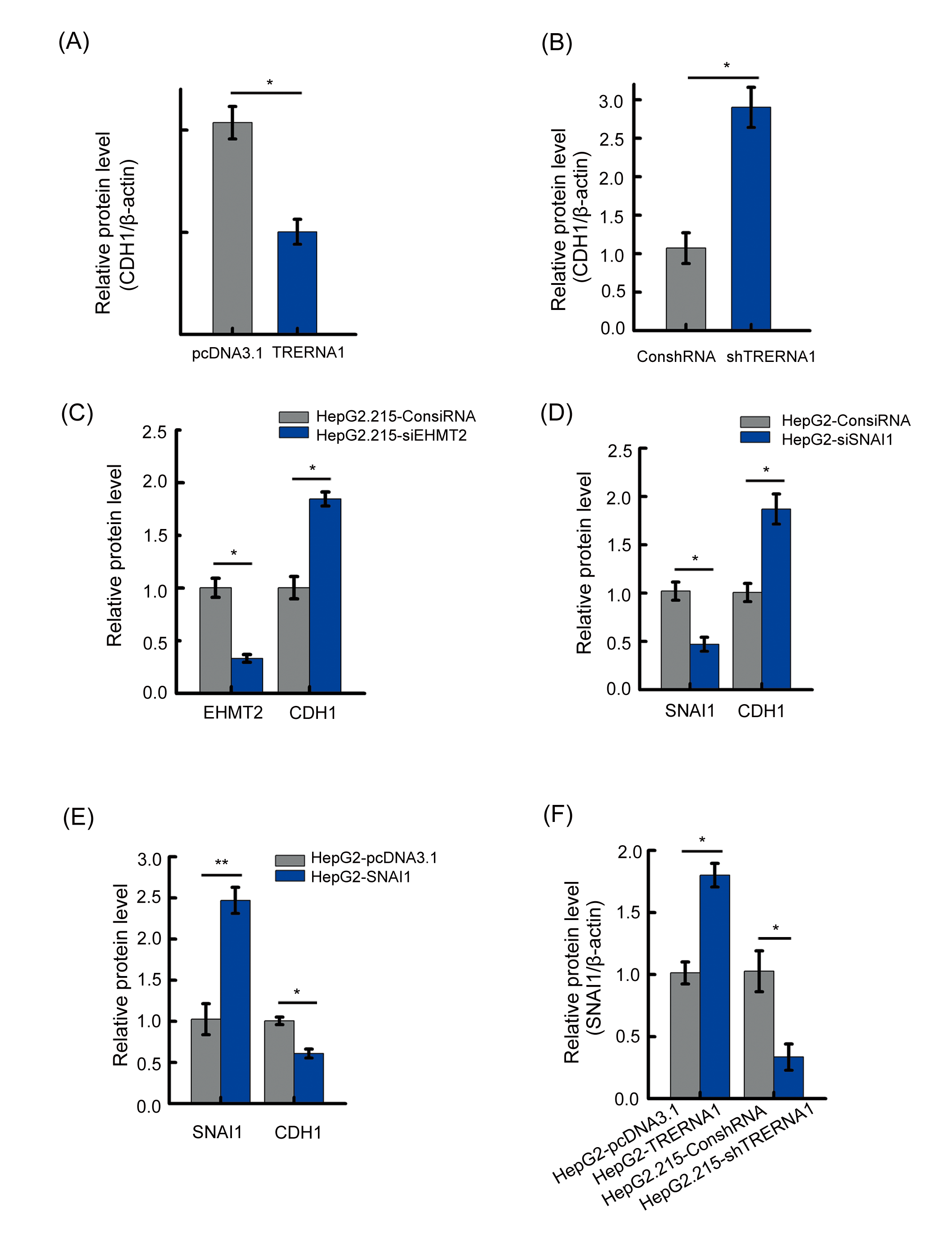

Supplement: Supplementary file 4 [file CPR-52-e12621-s004.tif]

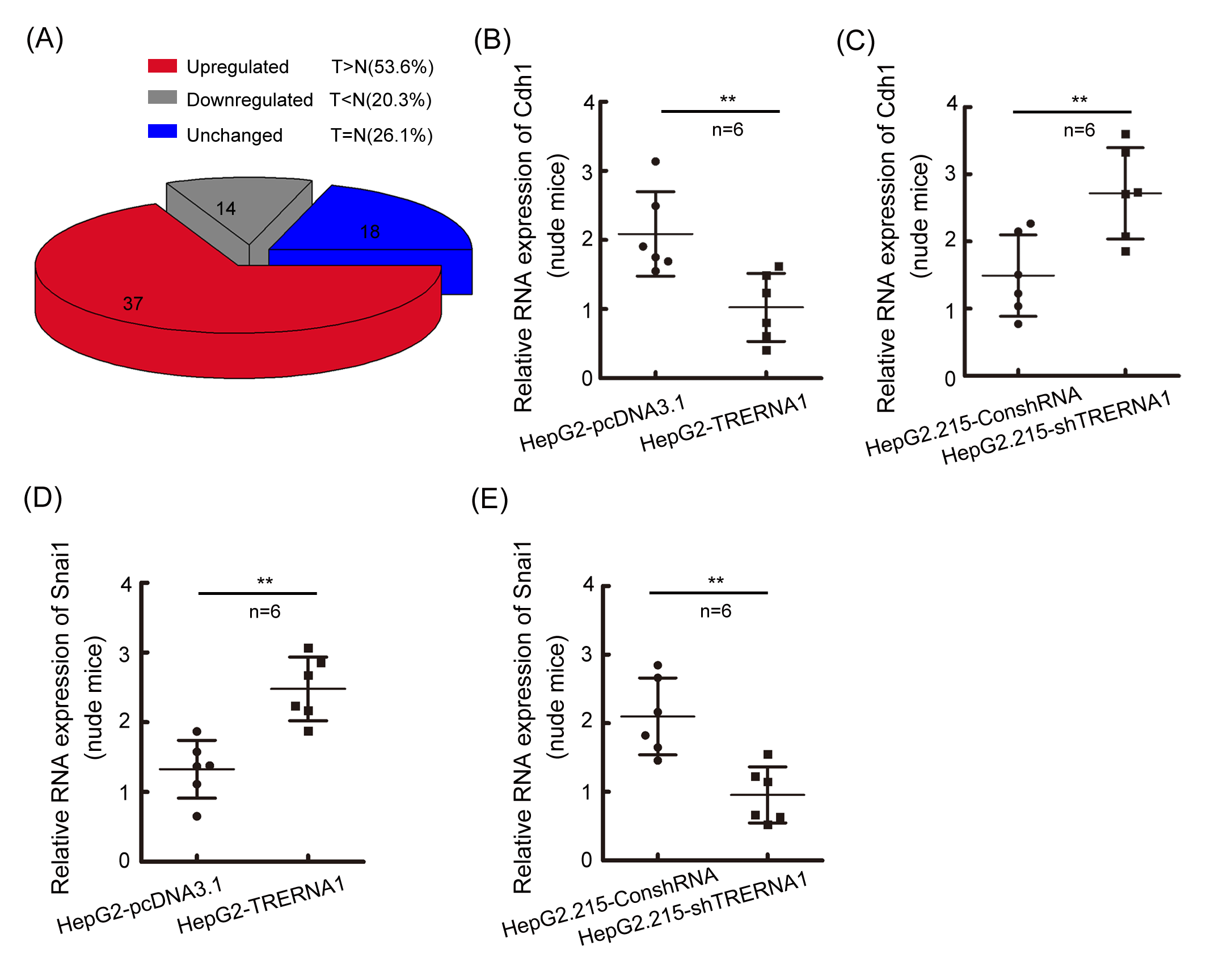

Supplement: Supplementary file 5 [file CPR-52-e12621-s005.tif]
