# Supplementary material for: LncRNA TRERNA1 facilitates hepatocellular carcinoma metastasis by dimethylating H3K9 in the CDH1 promoter region via the recruitment of the EHMT2/SNAI1 complex
Source: Cell Prolif. 2019 Apr 22;52(4):e12621. doi: 10.1111/cpr.12621 (PMC6668973; doi:10.1111/cpr.12621)
Supplement: Supplementary file 6 [file CPR-52-e12621-s006.docx]

**Supplementary Tables**

**Table S1** **siRNA sequence of genes used in this manuscript**

| Gene names Sequences | | |
| --- | --- | --- |
| Control-siRNA | Sense | GCUUCCUGCUACACAGAAUUU |
|  | Antisense | AUUCUGUGUAGCAGGAAGCUU |
| TRERNA1-siRNA1 | Sense | GAAGGGAACCAGUGCUAAAUU |
|  | Antisense | UUUAGCACUGGUUCCCUUCUU |
| TRERNA1-siRNA2 | Sense | CCGAUUUGAGAGAGUGAGAUU |
|  | Antisense | UCUCACUCUCUCAAAUCGGUU |
| SNAI1-siRNA | Sense | GAUGCACAUCCGAAGCCAC |
|  | Antisense | GUGGCUUCGGAUGUGCAUC |
| EHMT2-siRNA1 | Sense | CACACAUUCCUGACCAGAGAU |
|  | Antisense | AUCUCUGGUCAGGAAUGUGUG |
| EHMT2-siRNA2 | Sense | GCUCCAGGAAUUUAACAAGAU |
|  | Antisense | AUCUUGUUAAAUUCCUGGAGC |
|  |  |  |

**Table S2** **Primers used for reverse transcription and real-time PCR**

| Primer names Sequences | | |
| --- | --- | --- |
| TRERNA1 | Forward(5’-3’) | CCGTTGGCTCCACAAACCT |
|  | Reverse (5’-3’) | CAGTGACAGTAGCAGGCATCCT |
| β-actin | Forward(5’-3’) | GTCATTCCAAATATGAGATGCGT |
|  | Reverse (5’-3’) | GCTATCACCTCCCCTGTGTG |
| SNAI1 | Forward(5’-3’) | TTCAACTGCAAATACTGCAACAAG |
|  | Reverse (5’-3’) | TGTGGCTTCGGATGTGCAT |
| CDH1 | Forward(5’-3’) | CCTGGGACTCCACCTACAGA |
|  | Reverse (5’-3’) | TGTGAGCAATTCTGCTTGGA |
| EHMT2 | Forward(5’-3’) | CTCTACCGAACAGCCAAGAT |
|  | Reverse (5’-3’) | CTCAGCATCAGAGATCAGC |
| CDH2 | Forward(5’-3’) | ACAGTGGCCACCTACAAAGG |
|  | Reverse (5’-3’) | CCGAGATGGGGTTGATAATG |
| Claudin-7 | Forward(5’-3’) | GTAGCTTGCTCCTGGTAT |
|  | Reverse (5’-3’) | AACTCATACTTAATGTTGGTAGG |
| vimentin | Forward(5’-3’) | CCTGAACCTGAGGGAAACTAA |
|  | Reverse (5’-3’) | GCAGAAAGGCACTTGAAAGC |
| FN1 | Forward(5’-3’) | CAGTGGGAGACCTCGAGAAG |
|  | Reverse (5’-3’) | TCCCTCGGAACATCAGAAAC |
| U6 | Forward(5’-3’) | CGCTTCGGCAGCACATATACTA |
|  | Reverse (5’-3’) | CGCTTCACGAATTTGCGTGTCA |
| GAPDH | Forward(5’-3’) | GCACCGTCAAGGCTGAGAAC |
|  | Reverse (5’-3’) | TGGTGAAGACGCCAGTGGA |
| ChIP CDH1 SiteA | Forward(5’-3’) | TGTCCGCCCCGACTTGTCTCTC |
|  | Reverse (5’-3’) | GTCCTCTGGCCCCAGCCTCTCT |
| ChIP CDH1 SiteB | Forward(5’-3’) | ACAAAAAATCCCAAAAAACA |
|  | Reverse (5’-3’) | CATAGACGCGGTGACCCTCT |
| ChIP CDH1 SiteC | Forward(5’-3’) | ACTCCAGGCTAGAGGGTCACC |
|  | Reverse (5’-3’) | CCGCAAGCTCACAGGTGCTTTGCAGTTCC |
|  |  |  |
