# Supplementary material for: LncRNA TRERNA1 facilitates hepatocellular carcinoma metastasis by dimethylating H3K9 in the CDH1 promoter region via the recruitment of the EHMT2/SNAI1 complex
Source: Cell Prolif. 2019 Apr 22;52(4):e12621. doi: 10.1111/cpr.12621 (PMC6668973; doi:10.1111/cpr.12621)
Supplement: Supplementary file 7 [file CPR-52-e12621-s007.docx]

**Supplementary figure legends**

**Supplementary Table S1.** siRNA sequence of genes used in this manuscript

**Supplementary Table S2.** Primers used for reverse transcription and real-time PCR

**Supplementary Figure S1** The overexpression or interference efficiency of TRERNA1. A, qRT-PCR analysis showing the expression of TRERNA1 in HepG2 cells stably transfected with TRERNA1 constructs or with control vectors. B, qRT-PCR analysis showing the expression of TRERNA1 in HepG2.215 cells stably transfected with shTRERNA1 constructs or with control vectors. The results are presented as the mean ± SD; *n* = 3. ***P* < 0.01 (Student’s *t*-test).

**Supplementary Figure S2** TRERNA1 promotes the cell migration and invasion of HCC *in vitro*. A, Cell migration assays were performed using transwell assays in Huh7 and Hep3B cells. The average number of cells exhibiting migration from three random microscopic fields is presented in the histogram. B, Cell invasion assays were performed using Matrigel-coated transwell membranes. The average number of cells exhibiting invasion from three random microscopic fields is presented in the histogram. C, D, Wound healing assays in TRERNA1-overexpressing Huh7 cells and in TRERNA1-depleted Hep3B cells. The scratch was measured at 0, 24 and 48 h. Data are presented as the mean ± SD; *n* = 3. **P* < 0.05, ***P* < 0.01.

**Supplementary Figure S3** TRERNA1 regulates the expression of CDH1. A, qRT-PCR analysis showing the expression of TRERNA1 in Huh7 cells transiently transfected with TRERNA1 constructs or with control vectors. B, qRT-PCR analysis showing the expression of TRERNA1 in Hep3B cells transiently transfected with siTRERNA1 constructs or with control vectors. C, The relative mRNA expression levels of CDH1 were measured in TRERNA1-overexpressing Huh7 cells. D, The relative mRNA expression levels of CDH1 were measured in TRERNA1-depleted Hep3B cells. Data are shown as the mean ± SD; *n* = 3. **P* < 0.05, ***P* < 0.01 (Student’s *t*-test).

**Supplementary Figure S4** The band intensity analyses of the western blot results. A, The band intensities of CDH1 were quantified in HepG2 cells transfected with TRERNA1. B, The band intensities of CDH1 were quantified in HepG2.215 cells transfected with shTRERNA1. C, The quantification of EHMT2 and CDH1 protein levels in HepG2.215 cells transfected with siEHMT2 or with the negative control. D, The quantification of SNAI1 and CDH1 protein levels in HepG2 cells transfected with siSNAI1 or with the negative control. E, The quantification of SNAI1 and CDH1 protein levels in HepG2 cells transfected with SNAI1 or with the negative control. F, The quantification of SNAI1 protein levels after transfection with TRERNA1/shTRERNA1 or with the negative control. β-actin was used as a loading control. The results are presented as the mean ± SD; *n* = 3. **P* < 0.05, ***P* < 0.01 (Student’s *t*-test).

**Supplementary Figure S5** The expression level of TRERNA1 and the relationship between TRERNA1 and CDH1. A, The expression level of TRERNA1 was analyzed by qRT-PCR in 69 paired HCC and their matched nontumor tissues (pie chart). The number of cases is 37,14,18. B, The expression level of Cdh1 was analyzed in mice liver tissues in the control and in the TRERNA1-overexpressing groups. C, The expression level of Cdh1 was analyzed from mice liver tissues in the control and in the TRERNA1-depleted groups. D, The expression level of Snai1 was analyzed in mice liver tissues in the control and in the TRERNA1-overexpressing groups. E, The expression level of Snai1 was analyzed from mice liver tissues in the control and in the TRERNA1-depleted groups. The results are presented as the mean ± SD; *n* = 3. **P* < 0.05, ***P* < 0.01 (Student’s *t*-test).
